# Diagnostic efficiency of abattoir meat inspection service in Ethiopia to detect carcasses infected with *Mycobacterium bovis*: Implications for public health

**DOI:** 10.1186/1471-2458-10-462

**Published:** 2010-08-06

**Authors:** Demelash Biffa, Asseged Bogale, Eystein Skjerve

**Affiliations:** 1 Department of Food Safety and Infection Biology, Centre for Epidemiology and Biostatistics, Norwegian School of Veterinary Science. P.O. Box 8146, Dep, 0033 Oslo, Norway; 2 Faculty of Veterinary Medicine, Hawassa University, P.O. Box, 5, Hawassa, Ethiopia; 3 College of Veterinary Medicine, Nursing and Allied Health, Tuskegee University, Williams-Bowie 114, Tuskegee, AL 36088, USA

## Abstract

**Background:**

Bovine Tuberculosis (BTB) is a widespread and endemic disease of cattle in Ethiopia posing a significant threat to public health. Regular surveillance by skin test, bacteriology and molecular methods is not feasible due to lack of resource. Thus, routine abattoir (RA) inspection will continue to play a key role for national surveillance. We evaluated efficiency of RA inspection for diagnosis of *Mycobacterium bovis *infection and discussed its public health implications in light of a high risk of human exposure.

**Methods:**

The study was conducted in five abattoirs: Addis Ababa, Adama, Hawassa, Yabello and Melge-Wondo abattoirs. The efficiency of routine abattoir (RA) inspection was validated in comparison to detailed abattoir (DA) inspection, followed by culture and microscopy (CM) and region of difference (RD) deletion analysis. Diagnostic accuracies (with corresponding measures of statistical uncertainty) were determined by computing test property statistics (sensitivity and specificity) and likelihood estimations using web-based SISA diagnostic statistics software. Post-test probability of detecting TB infected carcasses was estimated using nomograms. Agreement between RA and DA inspections was measured using kappa statistics. The study was conducted and reported in accordance with standards for reporting of diagnostic accuracy (STARD) requirements. Both routine and detailed meat inspection protocols were performed on a subpopulation of 3322 cattle selected randomly from among 78,269 cattle slaughtered during the study period. Three hundred thirty seven carcasses identified through detailed meat inspection protocols were subjected to culture and microscopy; of the 337, a subset of 105 specimens for culture and microscopy were subjected to further molecular testing.

**Results:**

There was a substantial agreement between RA and DA inspections in Addis Ababa (Kappa = 0.7) and Melge-Wondo abattoirs (Kappa = 0.67). In Adama, Hawassa and Yabello abattoirs, the agreement was however poor (Kappa ≤ 0.2). RA inspection was able to detect only 117 of the total 3322 carcasses inspected (3.5%). The sensitivity (Sn) and specificity (Sp) of RA inspection were 28.2% (95/337) [95%CI: 23.4-33.0] and 99.3% (2963/2985) [95%CI: 99.0-99.6], respectively, when DA inspection was considered as reference test. When culture and microscopy (CM) was considered as reference test, the Sn and Sp of RA were 55.2% (58/105) [95%CI: 45.7-64.7] and 84.1% (195/232) [95%CI: 79.3-88.8]. RA inspection failed to detect 71.8% (242/337) and 44.8% (47/105) of TB infected carcasses as judged by DA inspection and CM, respectively. On the other hand, a much higher sensitivity of DA was obtained when CM and RD deletion analysis were considered as reference tests (96.3% (105/109) and 100.0% (24/24), respectively).

**Conclusions:**

The study results indicate that meat inspection protocols currently utilized in abattoirs are insufficient to detect the majority of TB lesions at the gross level. DA inspection protocols were demonstrated to improve the detection level by approximately 3-fold. The failure of current inspection techniques to detect approximately 70% of carcasses presented with grossly-visible lesions of TB at the slaughter-plants indicates the magnitude of meat-borne zoonotic TB as an on-going risk to public health. Standardization of abattoir inspection protocols (in line with international sanitary requirements), enhanced training and proficiency testing of meat inspections, and raising public awareness are recommended as essential and cost-effective interventions to improve meat inspection service in Ethiopia, with subsequent protection of consumers' health.

## Background

Bovine Tuberculosis (BTB) is one of the most important zoonotic diseases known to humans. WHO, in conjunction with FAO and OIE, recently classified BTB as a neglected zoonotic disease with special reference to developing countries [1]. Proximity between farmers and their cattle, the customs of consuming raw milk and meat, and the virtual absence of meat inspection services in slaughterhouses constitute a high risk of transmission to humans [2]. In African countries, though BTB is known to be endemic and the risk of transmission to humans is high, the precise role of *M. bovis *in the epidemiology of human tuberculosis has not been well defined. In countries where BTB is not controlled, in most cases young persons acquire infections through drinking contaminated milk leading to extra-pulmonary forms of tuberculosis [3]. Adult humans at occupational risk, especially farmers and abattoir workers as well as veterinarians, are at increased risk by the respiratory route through aerosols from infected cattle and are more likely to develop typical pulmonary tuberculosis [4]. The fact that eating raw or undercooked meat is one way of contracting BTB [5] has great implications for importance of BTB as a zoonotic disease in Ethiopia, since raw meat consumption is local cultural habit and because BTB is highly prevalent in the cattle population and control measures are not implemented.

The relatively high proportion of extra-pulmonary cases among Ethiopian TB patients [6] signifies the role of mycobacteria including *M. bovis*, and of non-respiratory pathways of exposure. In a recent study carried out in rural Ethiopia, it has been demonstrated that the state of proportion of extra-pulmonary TB cases due to *M. bovis *was greater than that of *M. tuberculosis *[7]. Kidane et al. [8] also reported *M. bovis *as a cause for 17.2% (6/35) of the cervical lymphadenitis cases from rural Ethiopia.

In diagnosis of mycobacterial infections, culture is still considered the international gold standard [9]. However, due to dysgonic and slow growth characteristics, the identification of *M. bovis *by culture and biochemical methods is cumbersome and time consuming [10]. Furthermore, application of molecular techniques is expensive as it demands availability of adequate laboratory resources and trained personnel. In Ethiopia, regular testing of millions of individual cattle for surveillance of BTB by the tuberculin skin test (TST) is both difficult and logistically unfeasible due to resource limitations. Therefore, abattoir inspection remains the only affordable technique for monitoring the prevalence of BTB in Ethiopia.

The primary reason for post mortem examination of carcasses at slaughterhouse is for protection of public health [11]. TB status of slaughter cattle provides useful information and is a proxy indicator for the prevalence of TB-positive slaughter animals, and therefore likelihood of the human exposure through consumption of infected meat. Apart from providing data for regulatory programmatic awareness of the true prevalence of TB infection, carcass examination also provides clues as to whether the infection is in its early stage or has reached the transmissible stage [11]. This provides better programmatic awareness with subsequent development of targeted guidance on how to reduce the risk of TB spread within the specific geographic area, as well as opportunities to trace back the source of infection to the herds.

Ethiopia has the largest livestock population in Africa, with an estimated 44.3 million cattle, 23.6 million sheep, 26.3 million goats and 2.3 million camels [12]. Ethiopia's increasing human population, coupled with expanding urbanization and higher average income is putting increasing pressure on the meat supply. To meet this demand, millions of food animals are slaughtered every year throughout the country. In 2007, for example, a total of 18.8 million cattle, sheep, goats and camels were slaughtered at municipal abattoirs, primarily for domestic consumption [13]. For this reason, close monitoring of meat hygiene, including proper implementation of meat inspection procedures during slaughter, should be a vital part of the national public health protection program. Our current work is a continuation of the work published earlier describing prevalence of BTB in Ethiopian slaughter cattle [14]. With generation of new additional data from laboratory work (culture and microscopy and molecular analysis), we wanted to evaluate how well abattoir meat inspection protocols in Ethiopia are performing to detect cattle infected with *M. bovis*. Public health implications of the findings are also discussed. Recommendations for improving the service within the context of the prevailing cultural and socio-economic situation were generated as an important outcome of these efforts.

## Methods

### The abattoirs

The study was carried out between August 2006 and January 2007 in five abattoirs: Addis Ababa, Adama, Hawassa and Yabello Municipal abattoirs, and Melge-Wondo Meat Export abattoirs. These abattoirs were selected based on their representativeness to the prevailing husbandry practices, geographic location, and proximity to the livestock markets and trade routes. Detail information of each abattoir was presented as follows:

#### Addis Ababa municipal abattoir

is established 60 years ago, located in the heart of Addis Ababa, the capital city, is the biggest abattoir in the country providing 50% of the daily beef requirements of the city's residents [15]. It is at present administered under Addis Ababa abattoir enterprises with involvement of the quarantine division of the federal ministry of agriculture in close supervision of the meat inspection activities. Most of the cattle slaughtered at the abattoir are adult males of local zebu, though lesser numbers of crossbred males and calves as well as culled dairy cows are also slaughtered [15]. In recent years, the abattoir's mission evolved from the previous sole provision of slaughter and inspection services to becoming independent business enterprise with the aim of financial self sufficient. Each day, on average 500 head of cattle plus considerable numbers of goats, sheep and occasionally pigs are slaughtered. The abattoir has adequate electric light and water supplies as well as facilities to dispose condemned carcasses and offals.

#### Adama municipal Abattoir

is located in Adama Township, 90 km southeast of Addis Ababa. Administered by the town municipality, Adama abattoir is a moderate-sized abattoir established to provide slaughter and inspection services to local butcher shops in the town. On average, 50 cattle, 50 sheep, 40 goats and 10 camels are slaughtered daily. The overall abattoir sanitary environment is below the requirements of good hygiene practices (GHP) in slaughterhouses. There are poor internal facilities and sanitary conditions. Neither place for disposal of condemned carcasses nor facilities for wastewater treatment exist. Three assistant meat inspectors deliver services in Adama.

#### Melge-Wondo meat export abattoir (MWMEA)

is the largest export abattoir in the country built over 50 years ago. It is located in the densely forested mountainous area of Wondogenet, 265 km south of Addis Ababa. The abattoir has adequate electric light, water supply and offal disposable facilities. The abattoir is part of the ELFORA Agro-Industries PLC, the largest Livestock Company in Ethiopia. MWMEA exports livestock and meat products to the Middle East countries (Saudi Arabia, UAE and Yemen) and to African Countries (Djibouti, Congo Brazzaville, Cotê-d'ivoire and Egypt). In recent years, MWMEA has been utilized exclusively for beef carcass export to Egypt, though occasional slaughter services are provided to meet local needs. One DVM, 2 meat inspectors and 1 assistant meat inspector have involved in meat inspection services at MWMEA.

#### Hawassa municipal abattoir

is located in Hawassa Township, 270 km south of Addis Ababa. It is administered by the town municipality and provides slaughter and inspection services for three butcher shops in the town. On average 50 cattle, 30 sheep and goats are slaughtered each day. The overall abattoir environment falls short of the standard level. It is operated by one junior veterinarian and three assistant meat inspectors.

#### Yabello municipal abattoir

located in vicinity of Yabello town, 575 km south of Addis Ababa, is virtually a rural small slaughterhouse far below the required standard. It doesn't have adequate supply of water. Offal disposal and electric supply facilities do not exist. On average, seven animals (mainly cattle) are slaughtered each day in Yabello, supplying beef to three butcher shops in the town. This is a very low figure in relation to the large cattle population in the area. Characterized predominantly by cattle pastoralism, the area is a leading supplier of live cattle to national and international markets. The abattoir is operated by one assistant meat inspector.

### Study animals

A total of 3322 carcasses were selected randomly from among 78,269 cattle slaughtered during the study period. The figures included 3094 (93.1%) indigenous Ethiopian zebu cattle, 140 (4.2%) cross-bred cattle (Local zebu × Holstein) and 88 (2.7%) Holstein cattle. Table 1 shows sex and age distribution of study animals by breed.

**Table 1:** Distribution of sex and age of animals by breed

	Sex			Age		
Breed	Male	Female	Total	****Young****^**1**^	**Old**^**2**^	Total
Local^a^	2,732 [88.3]	362 [11.7]	3,094	2,009 [64.9]	1,085 [35.1]	3,094
Cross-bred	117 [83.6]	23 [16.4]	140	56 [40.0]	84 [60.0]	140
Pure exotic^c^	27 [30.7]	61 [69.3]	88	53 [60.2]	35 [39.8]	88

Total	2,876 [86.6]	446 [13.4]	3,322	2118 [63.8]	1,204 [36.24]	3,322

^a^Indigenous Ethiopian zebu cattle; ^b^Local zebus cross with Holstein cattle; ^c^Holstein cattle. ^1^Young (< 6 years); ^2^Old (≥ 6 years).

The zebus are managed under traditional husbandry system (especially in pastoral/agro-pastoral areas) and depend entirely on natural pastures without extra feed supplements or veterinary services. Both pure and Holstein crossbred cattle belong to the state and private dairy farmers who are concentrated in central region of the country where commercially produced feed, veterinary and artificial insemination services are nearby.

### Routine abattoir inspection

Routine abattoir inspection was carried out by assigned meat inspectors in each abattoir. The procedures employ meat inspection protocols issued by the former Meat Inspection and Quarantine Division of the Ministry of Agriculture (MIQD) [16]. The procedure involves visual examination and palpation of intact organs such as the liver and kidneys, and palpation and incision of tracheobronchial, mediastinal and precrural lymph nodes. Further examinations of other lymph nodes and organs, or body system are considered whenever lesions are detected in one of these tissues. The whole carcass is condemned if miliary TB involving multiple lymph nodes is detected, while the whole organs (or their parts) are condemned if large tuberculous lesions are found in their parenchyma or associated lymph nodes [15]. Findings from routine abattoir meat inspection are recorded on meat inspection disease analysis report format. The system requires compilation of documents on daily, monthly and yearly disease records (by slaughterhouse, species of animals slaughtered, organ and part inspected and the findings, and decision made on the findings).

### Detailed meat inspection

A detailed meat inspection of each of the carcass was carried out by the principal investigator and his assistants together with assigned qualified experts inside the slaughterhouse and/or in the lab as necessary. In each carcass examined, the following lymph nodes were incised and inspected for presence of any lesions compatible with BTB: parotid, retropharyngeal, mediastinal, tracheo-bronchial, mesenteric, submaxilliary, iliac, precrural, prescapular, supramammary, inguinal, apical, ischiatic, portal and sternal lymph nodes. Organs/tissues including the lungs, liver, kidneys, mammary tissue, intestines, heart, abdominal and thoracic cavities and meninges were thoroughly examined (visually and by palpation as necessary) on the spot and/or collected and transported aseptically to the nearest laboratory for subsequent examination. In the laboratory, the lymph nodes were cut into slices of 2 mm; other organs were incised at intervals of 2 cm using a sterile surgical blade (24:4L Blade-Handler size ratio, BD Bard-Parker^TH ^Protected Blade System, UK); and cut surfaces were examined visually under a bright light source for the presence of TB like lesions [17]. Findings from detailed inspection were recorded for each tissue/organ on separate data recording sheet developed for this purpose.

### Sample collection and processing

Based on the routine and detailed abattoir inspection results, 406 tissue specimens belonging to 26 different organs/tissues were collected from 337 carcases with TB like granulomatous lesions for isolation and identification of mycobacteria. Organs/tissues collected include: tracheobronchial lymph nodes (ln) (n = 112); mediastinal ln (n = 89), apical ln (n = 45); retropharyngeal ln (n = 27); parotid ln (18); submaxilliary ln (n = 10); mesenteric ln (n = 103); portal ln (n = 10); ischiatic ln (n = 2); prescapular ln (n = 26); precrural ln (n = 21); supramammary ln (n = 6); inguinal ln (n = 1); sternal ln (n = 4); iliac ln (3); lungs (n = 85); liver (n = 29); kidneys (n = 9); spleen (6); mammary gland tissue (n = 2); intestinal tissues (n = 4); muscles (cardiac and ribs) (n = 4); samples from thoracic and abdominal cavities (n = 14); samples from vertebrae (n = 6); and meninges (n = 14).

For the purpose of comparison, samples from 14 carcasses randomly selected from lesion negative carcasses by detail abattoir inspection (n = 2985) were subjected to culture and microscopy (CM) and molecular differentiation. Samples were collected in sterile universal plastic bottles, kept in deep freezer, and later stored at -25°C in the tuberculosis laboratory of Ethiopian Health and Nutrition Research Institute (EHNRI), Addis Ababa, until transported to the BSL-3 laboratory of the National Veterinary Institute, Oslo, Norway for bacteriological analysis. Precautionary measures were taken during collection, handling and transportation of the specimens so as to avoid contamination and human exposure. Cross-contamination of specimens during collection and transportation was avoided by using different sets of sterile instruments and containers for each case sampled. Permission for transfer of the materials to Norway for detail laboratory analysis was obtained in advance from the Veterinary authorities of meat inspection and quarantine division of the Ethiopian Ministry of Agriculture and from the Norwegian Food Safety Authority.

Sample processing and culturing for isolation of mycobacteria was carried out at the mycobacteriological laboratory at the National Veterinary Institute, Norway following the standard procedure described in the manual of diagnostic tests and vaccines for terrestrial animals [18]. The samples were divided into two equal portions one for culturing and the other stored at -80°C to allow re-testing if necessary.

Briefly, about 50 g of sample was cut into pieces using a sterile scalpel blade and ground with addition of fine sand. Homogenization of the tissue specimen was carried out by gradual addition of 25 ml of saline water while grinding. Decontamination was carried out using 5% oxalic acid with subsequent incubation at room temperature for 15 minutes. Two drops of the sediment was then inoculated onto slant media of solid egg-based Lowenstein-Jensen (without pyruvate), Stone-brinks Medium (with glycerol) and agar-based Middlebrook 7H10. The inoculated media were then incubated aerobically at 37°C for at least 2 months with weekly monitoring for any mycobacterial growth. When growth was visible, smears were prepared and stained by Ziehl-Neelsen staining techniques. A colony from growth with positive microscopy (acid fast bacilli) was re-cultured on same sets of media so as to harvest pure culture. Characteristic growth patterns, morphology and colour of the colonies together with positive results for acid fast bacilli based on Ziehl-Neelsen technique were used for presumptive identification of mycobacteria [18].

### TB positive case definition by culture and microscopy (CM) and molecular method

Based on culture and microscopy, TB positive case was defined as carcass showing mycobacterial presence (by Ziehl-Neelsen staining and growth characteristics) in at least one sample collected from any part of the carcass. Where as case definition by molecular method was based on genomic region deletion analysis of the presence of *M. bovis *isolates obtained from at least one of the samples collected from a carcass. A total of 105 (25.9%) (out of 406 samples cultured) isolates were identified as being mycobacteria based on culture and microscopy. The isolates were further subjected to AccuProbe Gene Probe method for identification of mycobacteria belonging to *Mycobacterium tuberculosis *complex (MTC). The test procedures were carried out in accordance with the manufacturer's protocol. Briefly, a loopful of culture from Middlebrook 7H10 media was transferred into lysing reagent tube containing mixture of 100 μl each of cell lysis reagent and hybridization buffer. Cell lyses was performed by sonication for 15 min and subsequent heating at 95°C for 10 min. Hybridization of mycobacterial RNA with a DNA probe was carried out by transferring 100 μl of the lysed specimens into the probe reagent tubes and incubating at 60°C for15 min. The labelled DNA-RNA hybrids were measured in a GEN-PROBE luminometer. The results were interpreted as positive when samples produced signal ≥ 30,000 Relative Light Units (RLU), negative when < 20,000 RLU, and doubtful if between 20,000 - 29,000 RLU. Positive and negative control strains of *M. tuberculosis *and *M. avium *were obtained from the *Mycobacterium *culture collection of the National Veterinary Institute, Oslo, Norway.

Isolates positive for MTC Accu-probe test (n = 58) were subjected to region of difference (RD) deletion analysis for species identification of mycobacteria as described by Warren et al. [19]. Briefly, a multiplex hot start PCR was carried out with set of primers (RD1, RD4, RD9 and RD12). The mixture contained 12.5 μl of the hotstart redTaq, multiplex master mix (Sigma), 3.5 μl of the RNAse free water and 0.5 μl of each 50 pM primer. The reaction was run in PCR machine (GeneAmp^® ^PCR System 9700) at a denaturation temperature of 95°C for 15 min, and 40 cycles of 94°C for 1 min, 62°C for 1 min and 72°C for 1 min, with a final extension step at 72°C for 10 min and a holding step at 4°C. The PCR products were separated by electrophoresis (Bio-Rad sub-Cell^® ^Model 192) by running at 10 V/cm for 2 hours on 2% agarose gels. The resulting gel images were analyzed using gel imaging machine (Bio-Rad Gel Doc 2000). *M. bovis *was then differentiated from other MTC based upon the presence or absence of examined region of differences.

### Data collection

Data relating to individual animals (sex, age, breed, type/class) and management systems were collected on data recording sheets. Findings from routine abattoir inspection were obtained from meat inspection disease analysis reports from each abattoir. The number of staff involved in meat inspection in each abattoir was also recorded. Observations were also made regarding the existing facilities such as water and electric light supplies, and sewage and offal disposal facilities. Results from detail meat inspection, bacteriology and molecular method were recorded on Excel spread sheets for appropriate test comparison analysis.

### Methods for comparison of diagnostic tests

Efficiency of routine abattoir inspection to detect tuberculous carcasses (with detailed necropsy, bacteriology and molecular method as reference tests) was assessed based on computation of diagnostic accuracy measures (with corresponding measures of statistical uncertainty). The measures mainly include sensitivity, specificity, positive and negative predictive values. Further evaluation of the validity of routine abattoir inspection procedure was carried out by computing the likelihood ratio (LR). The kappa test was used to assess the degree of agreement between the tests. All the necessary diagnostic statistical analyses were carried out using the on-line software SISA (Simple Interactive Statistical Analysis-Diagnostic statistics) [20]. Similarly, the pre-and post-test infection probabilities were illustrated graphically by Fagan's nomogram using diagnostic test calculator software [21]. The study conforms to the international standards for reporting of diagnostic accuracy (STARD) requirements [22].

## Results

Table 2 presents the distribution of TB infected carcasses and efficiencies of routine abattoir inspection procedure in different abattoirs. There was a substantial agreement between routine and detailed meat inspection procedures at two abattoirs [Addis Ababa (Kappa = 0.7) and Melge-Wondo abattoirs (Kappa = 0.67)]. Sensitivity of finding TB + granulomatous lesions during routine meat inspection at these abattoirs was 60.0%. In the other three abattoirs however, the agreement between routine abattoir and detailed meat inspections was relatively poor (Kappa ≤ 0.2) indicating a much lower sensitivity. Table 3 presents the results of routine abattoir inspection versus detailed inspection procedure, culture and microscopy (CM) and molecular method for evaluation of the relative efficiency of routine abattoir inspection protocols for diagnosis of BTB in cattle in Ethiopia.

**Table 2:** Distribution of tuberculosis infected carcasses and test properties of routine abattoir inspection in different abattoirs in Ethiopia

	Test comparison			Test properties of routine abattoir inspection				
	
	Routine abattoir inspection	Detailed inspection		Kappa [95% CI]	Sn (%) [95% CI]	Sp (%) [95% CI]	PV+ (%) [95% CI]	PV-(%) [95% CI]
		+ve	-ve					
Addis Ababa	+ve	54	-	0.7 [0.7-0.72]	60.0 [50.0-60.0]	100.0 [99.0-100.0]	100.0 [93.0-100.0]	93.0 [92.0-93.0]
	-ve	37	509					
	Total	91	509					
	%	15.2						
Adama	+ve	4	-	0.05 [0.02-0.05]	3.0 [2.0-3.0]	100.0 [99.0-100.0]	100.0 [51.0-100.0]	76.0 [75.0-76.0]
	-ve	125	393					
	Total	129	393					
	%	24.7						
Melge-Wondo	+ve	36	9	0.67 [0.57-0.75]	60.0 [51.0-66.0]	99.0 [90.0-99.0]	80.0 [68.0-88.0]	98.0 [97.0-98.0]
	-ve	24	1256					
	Total	60	1265					
	%	4.5						
Hawassa	+ve	-	-	0.0 [0.0-0.0]	0.0 [0.0-0.0]	100.0 [100.0-100.0]	0.0 [0.0-0.0]	91.0 [91.0-91.0]
	-ve	39	403					
	Total	39	403					
	%	8.8						
Yabello	+ve	1	13	0.03 [-0.03-0.22]	6.0 [1.0-22.0]	97.0 [96.0-97.0]	7.0 [1.0-29.0]	96.0 [96.097.0]
	-ve	17	402					
	Total	18	415					
	%	4.2						

% = apparent prevalence based on detailed abattoir inspection results

**Table 3:** Evaluation of diagnostic accuracies (with corresponding 95% CI) of routine abattoir inspection to detect carcasses infected with *M. bovis *in Ethiopia (detail abattoir inspection, culture and microscopy and molecular method considered as reference tests)

Test properties		Detailed inspection			Culture and microscopy			Molecular method		
		
		+ve	-ve	Total	+ve	-ve	Total	+ve	-ve	Total
Routine abattoir inspection	+ve	95	22	117	58	37	95	15	43	58
	-ve	242	2963	3205	47	195	242	9	38	47
										
	Total	337	2985	3322	105	232	337	2 24	81	105

Sn (%) [95% CI]		28.2 [23.4-33.0]			55.2 [45.7-64.7]			62.5 [43.1-81.9]		
Sp (%) [95% CI]		99.3 [99.0-99.6]			84.1 [79.3-88.8]			46.9 [36.0-57.8]		
PV+ (%) [95% CI]		81.2 [74.1-88.3]			61.1 [51.2-70.9]			25.9 [14.6-37.1]		
PV- (%) [95% CI]		92.4 [87.7-93.0]			80.6 [72.6-88.5]			80.9 [70.7-91.0]		
LR+[95% CI]		38.3 [24.4-59.9]			3.5 [2.5-4.9]			1.2 [0.8-1.7]		
LR-[95% CI]		0.72 [0.68-0.77]			0.53 [0.43-0.66]			0.8 [0.45-1.4]		
Kappa [95% CI]		0.39 [0.35-0.42]			0.44 [0.2-0.45]			0.1[-0.10.2]		

Out of 3,322 carcasses examined, routine abattoir inspection identified only 117 (3.5%) carcasses with gross pathologic lesions compatible with TB; where as detailed meat inspection procedures identified 337 (10.2%) carcasses with TB lesions, a more than three fold difference. When compared with detail abattoir inspection, routine abattoir inspection failed to detect 71.8% (242/337) of TB infected carcasses with gross pathologic lesions (false negative), but only misclassified a negligible proportion (0.7%) (22/2985) as TB infected when they were not (false positives). The sensitivity (Sn) of routine abattoir inspection was therefore quite poor at only 28.2% [95% CI: 23.4-33.0]. However, the specificity (Sp) (for lesions identified to be TB) was very high at 99.3% [95% CI: 99.0-99.6]. Kappa test demonstrated a relatively fair agreement between the two abattoir inspection protocols (Kappa = 0.39 [95% CI: 0.35-0.42]).

When culture and microscopy was utilized as a reference test, the Se of routine abattoir inspection was 55.2% ([95% CI: 45.7-64.7] and Sp was 84.1% [95% CI: 79.3-88.8]. Routine abattoir inspection misclassified 44.8% (47/105) of mycobacterial infected carcasses as non-infected, and 15.9% (37/232) of non-infected as infected.

**Figure 1:**
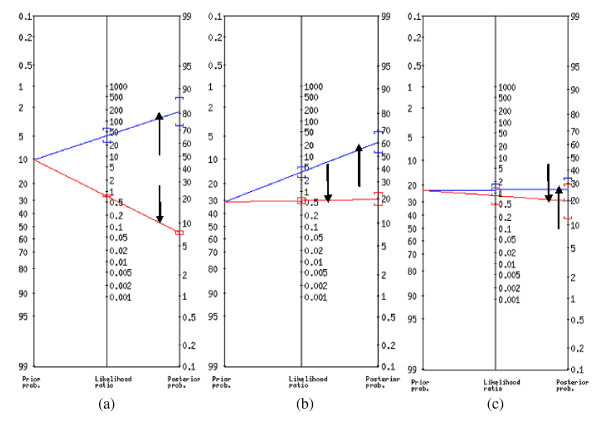
**Fagan's nomogram for post-test probabilities of TB occurrence in Ethiopian slaughter cattle based on routine abattoir inspection (comparisons were made with detailed abattoir inspection (a), culture and microscopy (b) and molecular method (c)**. ↑ = Positive outcome: ↓ = Negative outcome.

Figure 1 shows nomographic depiction to estimate post-test probability of TB infection from pre-test probability and likelihood ratio. According to the results, the pre-test probability (prior probability) of TB was estimated to be 10.0%, 23.0% and 20.0%, respectively based on detailed abattoir inspection, culture and microscopy and molecular method. The post-test probability of tuberculous carcasses (predictive value for positive result of routine abattoir inspection) was found to decline to 81.0% when compared to detailed abattoir inspection and to 60.0% when compared to culture and microscopy (CM).

Efficiency of detailed abattoir inspection for diagnosis of *M. bovis *was established for subsample of carcasses inspected (351 for culture and microscopy (CM) and 119 for molecular method) as presented in Table 4. Culture and microscopy results of samples from 337 TB suspected carcasses were compared with samples from 14 lesion negative carcasses (by detailed abattoir inspection protocol). Detailed abattoir inspection identified the majority of carcasses as harbouring mycobacteria (105/109). Few carcasses (3.7%; 4/109) harbouring mycobacteria were not detected by DA. The sensitivity and specificity of detailed abattoir inspection were 96.3% (95% CI: 92.8-99.9) and 4.1% (95% CI: 1.6-6.6) when compared with results obtained from culture and microscopy. Detailed abattoir inspection identified all the 24 carcasses infected with *M. bovis*. The sensitivity and specificity of detail abattoir inspection was therefore, 100.0% and 14.7 (95% CI: 7.6-21.9). With molecular method taken as reference test, detailed abattoir inspection erroneously classified 85.3% (81/95) of carcasses as infected with *M. bovis *(Table 4).

**Table 4:** Evaluation of diagnostic accuracies (with corresponding 95% CI) of detailed abattoir inspection procedure to detect carcasses infected with *M. bovis *(culture and microscopy and molecular method considered as reference tests)

Test properties of Detailed inspection	Culture and microscopy			Molecular method		
	
	+ve	-ve	Total	+ve	-ve	Total
+ve	105	232	337	24	81	105
-ve	4	10	14	-	14	14
Total	109	242	351	24	85	119

Sn (%) [95% CI]	96.3 [92.8-99.9]			100.0 [100.0-100.0]		
Sp (%) [95% CI]	4.1 [1.6-6.6]			14.7 [7.6-21.9]		
PV+(%) [95% CI]	31.2 [26.2-36.1]			22.9 [14.8-30.9]		
PV-(%) [95% CI]	71.4 [66.6-76.3]			100.0 [100.0-100.0]		
LR+	1.0 [0.9-1.1]			1.2 [1.1-1.3]		
LR-	0.9 [0.3-2.8]			0.0 [0.0-0.0]		
Kappa [95% CI]	0.003 [-0.01-0.03]			0.1 [0.001-0.13]		

## Discussion

Because presence of gross pathologic lesions was the basis for selection of very small subsets of total carcasses in this study for additional culture and microscopy (337/3322) or molecular testing (105/3322), it is likely that the incidence of BTB in these carcasses represents a very conservative under-estimate of the actual incidence of TB-positivity in this cattle population.

At the abattoir level, routine inspection missed all infected carcasses in Hawassa abattoir compared to moderate numbers missed at Addis Ababa and Melge-Wondo (Table 2). This indicates a lack of standardization in the delivery of meat inspection service as reflected by the difference in the number and competence of skilled workers. Such variation in misclassification of cases of TB may also be attributable to the difference in experience and training of the workforce, particularly in small abattoirs where more experienced inspectors are not routinely present to provide supervision and monitoring. Misclassification may be attributable to the nature of the disease pathology (gross versus microscopic lesions), and may possibly be influenced by differences in livestock management systems (extensive vs. intensive production). In previous work, we demonstrated that TB lesion localization in specific tissues was a common finding in Hawassa; where as disseminated (miliary) forms were common in Addis Ababa and Melge-Wondo abattoirs [14].

Smaller (and fewer) localized tubercle lesions can easily be missed by routine meat inspection procedures because of the limited time spent in examining tissues and the difficulty in fine-slicing lymph nodes in situ [23]. Variations in the appearance of TB lesions within carcasses can make detection of lesions difficult especially for inexperienced inspection personnel [24].

Nearly 1% (9/1265) and 3% (13/415) false positive results were obtained from Melge-Wondo and Yabello abattoirs, respectively (Table 1). Such errors may be attributable to misdiagnosis of cattle TB with other granulomatous diseases such as Contagious Bovine Pleuropnuemonia (CBPP) and chronic pneumonic pasturellosis. The majority of cattle slaughtered in the two abattoirs were of the Boran zebu genotype with origin linked to Borena rangeland in South-eastern Ethiopia, where CBPP remains as a major endemic disease of cattle. CBPP often exhibits similar clinical pathology with bovine TB with predominant lesions observed in lungs [25].

In this study we evaluated the efficiency of routine abattoir inspection service in Ethiopia to detect carcasses infected with *M. bovis *(Table 3). Of the 3,322 carcasses examined by routine abattoir inspection, 3.5% (117/3322) were found to have lesions suggestive of TB. When detailed meat inspection procedure was applied, the proportion of positive carcasses increased to 10.1% (337/3322). Routine abattoir inspection was much less effective, detecting only 95 of the lesioned carcasses. The sensitivity and specificity of routine abattoir inspection was 28.2% ([95% CI: 23.5-33.0]) and 99.3% ([95% CI: 99.0-99.6]), respectively (Table 3). A similar finding (29.4% sensitivity) was previously recorded at Hosanna Municipal abattoir in Southern Ethiopia [26]. Both the Hosanna study and our current study have demonstrated a low efficiency of current abattoir inspection protocol, contrary to studies reporting that the procedure can detect 95% of carcasses with TB lesions [23]. Similarly, routine abattoir inspection failed to detect 44.8% (47/105) of infected carcasses (as confirmed by culture and microscopy) and 37.5% (9/24) (confirmed by molecular method) (Table 3). On the other hand, routine abattoir inspection recorded only 15.9% (37/232) and 53.1% (43/81) false positive carcasses (with culture and microscopy and molecular method considered, respectively as test comparison) (Table 3).

According to the results from analysis of retrospective data (1996-2008) from three major abattoirs across the country (Addis Ababa, Adama and Melge-Wondo), whole carcass condemnation rates attributable to TB infection were respectively 0.043%, 0.4% and 0.2%) [14]. This illustrates a significant underestimation of the occurrence of BTB in Ethiopia, mainly due to lack of standardized training and competency testing in proper (international standard) abattoir inspection procedures. In Ethiopia, because of endemic and widespread nature of TB in cattle linked to absence of control measures and high risk of transmission, one should anticipate a higher TB prevalence in slaughter cattle than current estimates based on routine abattoir inspection. Our study demonstrated that the routine abattoir inspection failed to detect a high proportion (72.0%) of lesioned carcasses identified by detail abattoir inspection (Table 3); such meats were erroneously passed as fit for human consumption. In Chad, where BTB control measures are not practised, abattoir meat inspection is able to demonstrate a 9% prevalence of TB in slaughter cattle [27]. In Mexico as well, meat inspection at slaughterhouses demonstrated a 16% prevalence of TB lesions [28]. These figures are much higher than infection rates reported from routine abattoir inspections in Ethiopia signifying the existence of substantial differences in effectiveness of abattoir inspection services between these countries. This situation puts consumers at a greater risk of acquiring zoonotic TB through ingestion of TB infected meat and milk.

Among the several major reasons which likely are contributing to low effectiveness of routine abattoir inspection service in Ethiopia are: 1) inadequately trained and competency-tested personnel; 2) lack of regular competency testing of meat inspectors; 3) heavy work loads, and 4) poor physical facilities at abattoirs such as inadequate light sources for inspection of carcasses during the night. The existing Ethiopian meat inspection proclamation (adapted in 1970, No.274/1970, amended: No.81/1970) demands inspection of all carcasses at slaughterhouses. Due to the aforementioned reasons, many of the guidelines under the proclamation are not enforced at abattoirs that produce meat for local consumption [29]. Routine abattoir meat inspection involves the use of manual developed by Meat Inspection and Quarantine Division of the Ministry of Agriculture (MIQD). It advises visual examination, palpation of organs and palpation and incision of lymph nodes associated with head, lungs and pleural cavity [16]. However, carcass inspection for detection of BTB in most abattoirs in Ethiopia involves very brief examination of fewer tissues and organs than stated in the guidelines. At present a workforce of 260 senior and junior meat inspectors and their veterinary supervisors are providing inspection service for a total of 171 slaughterhouses, slaughter slabs and export abattoirs throughout the country [30]. In 2007, about 18.8 million animals (cattle, sheep, goats and camels) were officially recorded as slaughtered throughout the country [13] (an average of 198 animals inspected by each inspector each day). Because of this heavy workload and the relatively rudimentary facilities which exist in many places, fewer tissues and organs than stated in the guidelines are inspected, and lesser time is spent on each carcass.

Furthermore, in most abattoirs in Ethiopia, animals are slaughtered during the night to provide consumers with fresh raw meat the next morning. In most rural slaughterhouses where the electric power supply doesn't exist or is inadequate, proper detection of TB lesions may not be possible, thus allowing more infected carcasses to pass unnoticed. Approval of infected meat for human consumption partly also emanates from weak control over the meat inspector's decision process during slaughtering. With an intention to avoid financial loss linked to carcass/organ condemnation, butchers understandably will attempt to influence meat inspectors regarding any decision to condemn carcasses with few or unapparent lesions.

The issue of zoonotic TB through consumption of raw/undercooked meat is a subject of debate. For example, the Food Safety Authority of Ireland [31] has recently stated that consumption of fresh meat poses a low health risk for TB owing to uncommon occurrence of viable pathogens in muscle mass in the Irish cattle population. However the fact that *M. bovis *is reported to have been isolated from muscles [32] and *M. bovis *is well-established as an occupational health risk for abattoir workers in countries where it has not been eliminated [33] could undermine the credibility of such an opinion, particularly in countries such as Ethiopia with a high prevalence of TB in cattle and high prevalence of HIV-TB co-infection in the human population. Kazwala et al. [34] reported that TB-contaminated meat poses a great public health danger in Tanzania particularly among pastoral communities who normally consume undercooked meat. In support of this argument, Acha and Szyfres [35] and Cosivie et al. [36] indicated a well-established risk of acquiring zoonotic TB through meat consumption in developing countries.

In Ethiopia, apart from customary red beef, organs such as liver and kidneys can pose a high health risk as they are often consumed raw. These organs form a localized circuit for tubercle bacilli dissemination and may not show any evidence of infection upon routine post mortem examination [31]. Unfortunately, the majority of Ethiopians living in the countryside are not well aware of the risk of zoonotic TB. In a study carried out in central Ethiopia [37], only 18.1% of the interviewed cattle owners knew that meat may be a vehicle for *M. bovis *transmission although 90% of the interviewees reported that they consumed both raw and cooked meat. Due to economic disparities, meat consumption is more frequently practised among urban than rural dwellers. In town restaurants, mainly raw beef known as *"kurt" *(fresh red beef meat) or *"kitfo" *(finely chopped beef mixed with traditional spices) are frequently served. In rural areas raw meat consumption is not habitually practised and low economic income in majority of rural households leads to weak purchasing power of meat or meat animals. This difference in meat consumption habit would give more likely chance of exposure of urban dwellers to meat-borne zoonotic TB. On the other hand, acquisition of zoonotic TB through aerosol exposure and/or milk consumption is higher in rural areas where humans and animals live in close proximity and raw milk consumption from household cows is a usual habit.

Zoonotic TB among abattoir workers in Ethiopia has never been highlighted as occupational disease and therefore remains unknown to the public. Inhalation exposure during slaughter procedures and ante-mortem inspection when infected carcasses are opened currently pose important, although undefined, risks for zoonotic TB transmission among the abattoir workers in Ethiopia.

Detailed meat inspection protocols for detection of TB lesions was evaluated; it showed higher sensitivity (96.3 [95% CI: 92.8-99.9] for detection of TB lesions although the positive predictive value was low (31.2 [95% CI: 26.2-36.1] (Table 4). As might be expected, some carcasses identified as TB-positive on the basis of gross lesions did not produce mycobacteria upon culture of tissue samples. At the sample level, of the 406 pathologic samples analyzed, 105 (25.9%) yielded mycobacteria (based on culture and microscopy). Out of these, 58 (55.2%) samples with *M. bovis *were identified using PCR-based deletion analysis of genomic region. Forty-nine non-tuberculous mycobacteria (NTM) isolates were identified from the same samples using 16 S rRNA gene sequencing and INNO-LiPA assay (detail analysis of NTM and their implication in human health is presented in an other paper: Biffa et al: unpublished).

One additional reason for not recovering mycobacteria from pathologic specimens may be the extended period (time) between collection of specimens and submission to the laboratory for analysis, which has been shown to reduce the likelihood of successful bacterial isolation [10]. In calcified and caseous necrotic granulomas, tubercle bacilli could die as part of the tissue necrotic process thus making cultivation and bacterial growth a low-yield task. Cousins et al. [24] suggest that multiplication of bacilli tends to be inhibited in lesions containing caseous exudates. Granulomatous lesions can be caused by other infectious agents such as fungi, *Staphylococcus*, *Actinomyces*, and *Actinobacillus *spp. by foreign bodies, and by the presence of inspissated and calcified pus, as common with *Actinomyces *[24]. Finally, the possibility that certain proportion of mycobacterial organisms may be killed by disinfection [28] and decontamination using strong oxalic acid as part of the sample processing for culture can not be ruled out. It is also possible that many of the carcasses may be infected with mycobacteria other than *M. bovis *that would not replicate in the *M. bovis*-selective culture medium [28] or other pathogens with different culture growth requirements [38]. Insufficient isolation of *M. bovis *from samples originating from TB suspected carcasses have been reported in Ethiopia [39] and other African countries [38,40,41].

## Conclusions

This study demonstrated the limited capacity of current abattoir meat inspection procedures in Ethiopia to detect carcasses infected with *M. b*ovis. When compared to detailed abattoir inspection protocols, routine abattoir inspection failed to detect the majority of infected carcasses (as confirmed by culture and microscopy). Based on five slaughter facilities in this study, these erroneous decisions result in approval of TB-infected meat for human consumption, not only at small local abattoirs, but even at major facilities supplying the capital city and export meat markets. In Ethiopia and other countries where dietary preferences mean that a significant proportion of meat is customarily consumed raw, lack of effective slaughter inspection protocols represents significant regulatory gap. Thus meat borne zoonotic TB continues to be an on-going and an important threat to public health in a nation that has significant populations of vulnerable HIV-infected citizens. The high TB prevalence in Ethiopian slaughter cattle (Table 2) provides useful information for use by public health and agricultural officials. It may be used as a proxy indicator of the level of TB prevalence in cattle populations and food-borne exposure of human populations. The significant differences in effectiveness of abattoir meat inspection staff across five abattoirs in different geographic areas likewise provide a useful information for regulatory and inspection programs. On the basis of findings of the present study, the following measures are recommended to improve meat inspection protocols currently in place with the aim of significantly reducing human exposure to zoonotic TB:

1) The 1970 meat inspection proclamation should be revised and updated in line with trends of changing disease situations in both humans and animal populations;

2) Establishment of standard occupational health and safety measures and facility requirements are needed to protect health of abattoir workers;

3) Public health information campaigns are needed to raise community awareness about the risk of TB transmission through consumption of raw/undercooked meat;

4) Provision of rotating supervisory staff to improve meat inspection protocols and reduce the likelihood of inappropriate influence by the butchers on local inspectors especially in small rural abattoir facilities and;

5) Organize regular capacity building through in-service training for both professionals (technical training) and zoonotic diseases awareness training for non-professional personnel working in abattoirs.

## Competing interests

The authors declare that they have no competing interests.

## Authors' contributions

DB: collected the data, run the lab works and prepared the manuscript, AB: field supervision, assisted manuscript preparation and critical revision; ES: project planning, acquisition of funds, assisted preparation and critical review of the manuscript. All authors have read and approved the final manuscript.

## Pre-publication history

The pre-publication history for this paper can be accessed here:

http://www.biomedcentral.com/1471-2458/10/462/prepub
